# Stüve–Wiedemann syndrome with a novel variant in the *LIFR* gene: A case report

**DOI:** 10.1097/MD.0000000000041342

**Published:** 2025-01-31

**Authors:** Hanan Sakr Sherbiny, Jaber A. Alfaifi, Anees Obaid Ahmed, Hany Hassan, Raydaa Abdullah, Shaher A. Alsuwat, Raghad M. Al-Juaid, Aseel A. Neyaz, Mohammed A. M. Oshi, Naglaa M. Kamal

**Affiliations:** a Department of Child Health, College of Medicine, University of Bisha, Bisha, Saudi Arabia; b Maternity and Children’s Hospital, Bisha, Saudi Arabia; c College of Medicine, University of Bisha, Bisha, Saudi Arabia; d Pediatric department, Alhada Armed Forces Hospital, Taif, Saudi Arabia; e College of Medicine, Taif University, Taif, Saudi Arabia; f Neurology Division, Gaafar Ibnauf Children’s Emergency Hospital, Khartoum, Sudan; g Pediatric department, Kasr Alainy Faculty of Medicine, Cairo University, Cairo, Egypt.

**Keywords:** dysautonomia, *LIFR* gene, skeletal dysplasia, Stüve–Wiedemann syndrome

## Abstract

**Rationale:**

Stüve–Wiedemann syndrome (SWS) is a rare, severe autosomal recessive disorder (#OMIM 601559) caused by pathogenic variants in the *LIFR* gene. It is characterized by skeletal dysplasia and dysautonomia and carries a high mortality rate in infancy, which decreases significantly after the age of 2. Detailed case descriptions enhance understanding of this rare condition.

**Patient concerns:**

We report a male, full-term infant born to consanguineous Yemeni parents with no family history of genetic disorders. Prenatal ultrasound revealed short, bowed long bones suggestive of skeletal dysplasia. At 12 hours of age, the infant developed respiratory distress, poor sucking, and an agitated cry. At 48 hours, he experienced unexplained hyperthermia, and a comprehensive septic workup was negative.

**Diagnoses:**

Initial findings included generalized hypotonia, hyporeflexia, and dysmorphic features (micrognathia, camptodactyly, short, and bowed limbs). Radiographic imaging revealed skeletal abnormalities. Whole exome sequencing identified a novel homozygous pathogenic variant in the *LIFR* gene (c.2257dup p.(Arg753Lysfs*20)), confirming the diagnosis of autosomal recessive SWS type 1.

**Interventions:**

The infant was admitted to the neonatal intensive care unit, received nasal oxygen support, and was managed with orogastric tube feeding due to poor sucking and swallowing.

**Outcomes:**

At 5 months, the infant remains dependent on orogastric tube feeding, with less frequent hyperthermic episodes.

**Lessons:**

SWS is a rare genetic disorder with a wide phenotypic spectrum. Early recognition and multidisciplinary management are crucial to addressing the high mortality risk associated with dysautonomia in infancy. Case reports of novel variants contribute to a deeper understanding of SWS and highlight the importance of tailored clinical care for improved outcomes.

## 1. Introduction

Stüve–Wiedemann syndrome (SWS) is a rare genetic disorder first described in 1971 by Stüve and Wiedemann,^[1]^ who identified the condition in 2 sisters with congenital bowed legs and camptodactyly, both of whom developed respiratory distress and died in the neonatal period. SWS follows an autosomal recessive inheritance pattern and is caused by pathogenic variants in the *LIFR* gene, located on chromosome 5p13. This was confirmed by Dagoneau et al^[2]^ in 2004. Clinically, SWS is marked by a combination of skeletal dysplasia and dysautonomia, particularly affecting the ciliary neurotrophic factor pathway.^[3]^ A meta-analysis of 69 cases highlighted the syndrome’s natural history, which includes high mortality in the first 2 years of life, followed by a significant decrease in mortality as dysautonomia symptoms regress.^[4]^ While the syndrome was once universally considered lethal in infancy, cases of long-term survival have since been documented.^[5,6]^ The genetic and phenotypic heterogeneity of SWS has been recognized, with some patients exhibiting full clinical symptoms without detectable genetic abnormalities, while others display incomplete phenotypic presentations, such as dysautonomia without skeletal dysplasia.^[7,8]^ A detailed understanding of SWS is necessary to improve early diagnosis and management, which can lead to better outcomes.

This report presents a full-term boy who exhibited skeletal dysplasia and dysautonomia symptoms, including hyperthermia, feeding difficulties, and respiratory distress. The diagnosis of SWS was suggested by characteristic radiological findings and confirmed by whole exome sequencing, which revealed a novel pathogenic variant in the *LIFR* gene.

## 2. Case scenario

**Figure 1.**
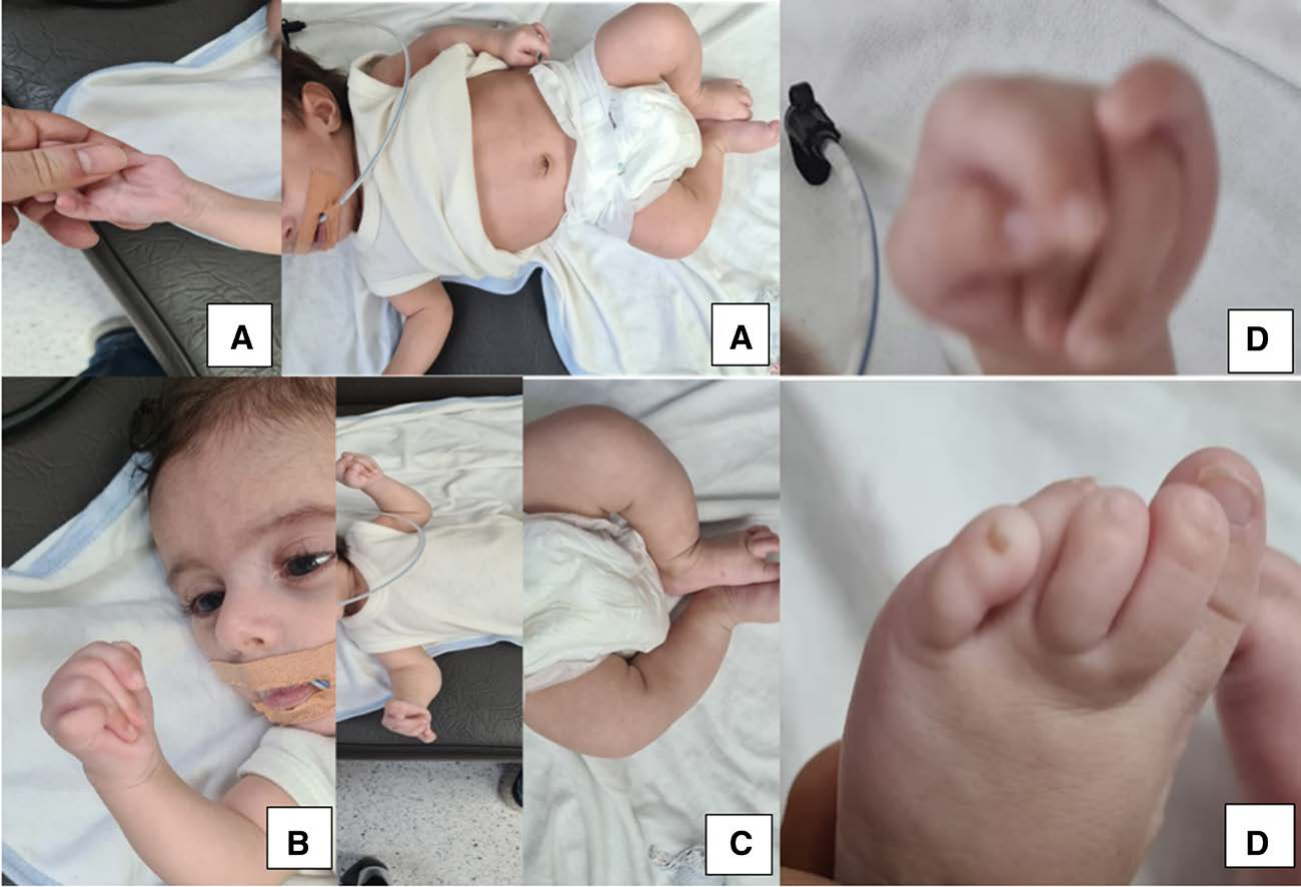
Phenotypic picture of the skeletal deformities. (A) Fixed joint contracture. (B) Micrognathia withorogastric tube in place. (C) Bowed short extremities. (D) Camptodactyly in fingers and toes.

**Figure 2.**
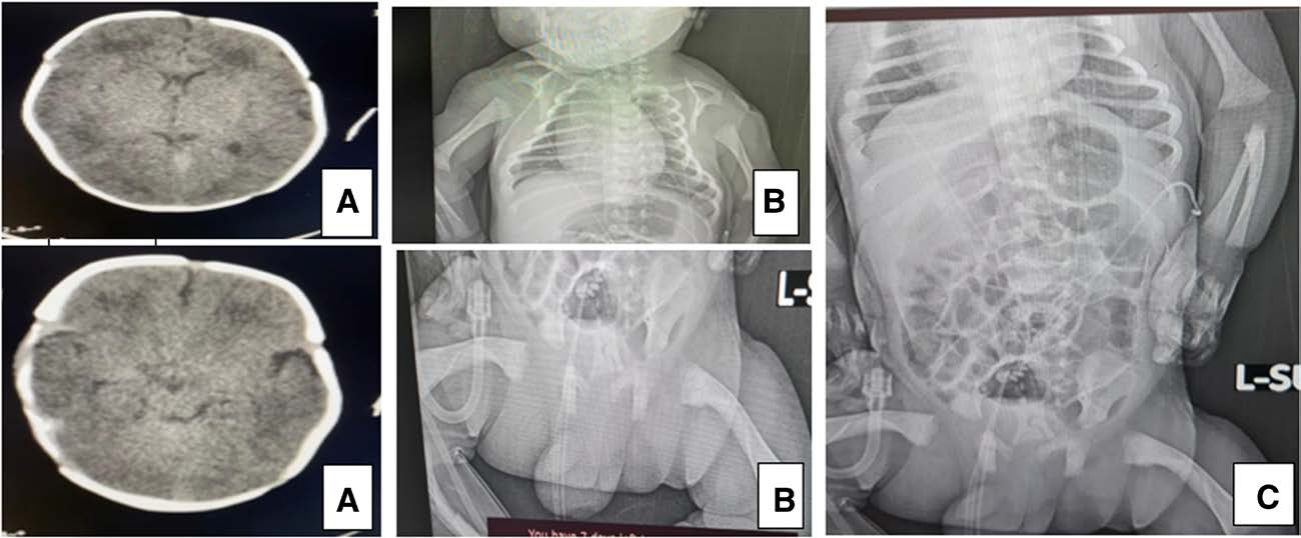
Radiologic findings ot the patient. (A) Bilateral periventricular hypodense areas. (B) Bilateral short bowed long bones. (C) Skeletal survey with short bowed long bones.

**Figure 3.**
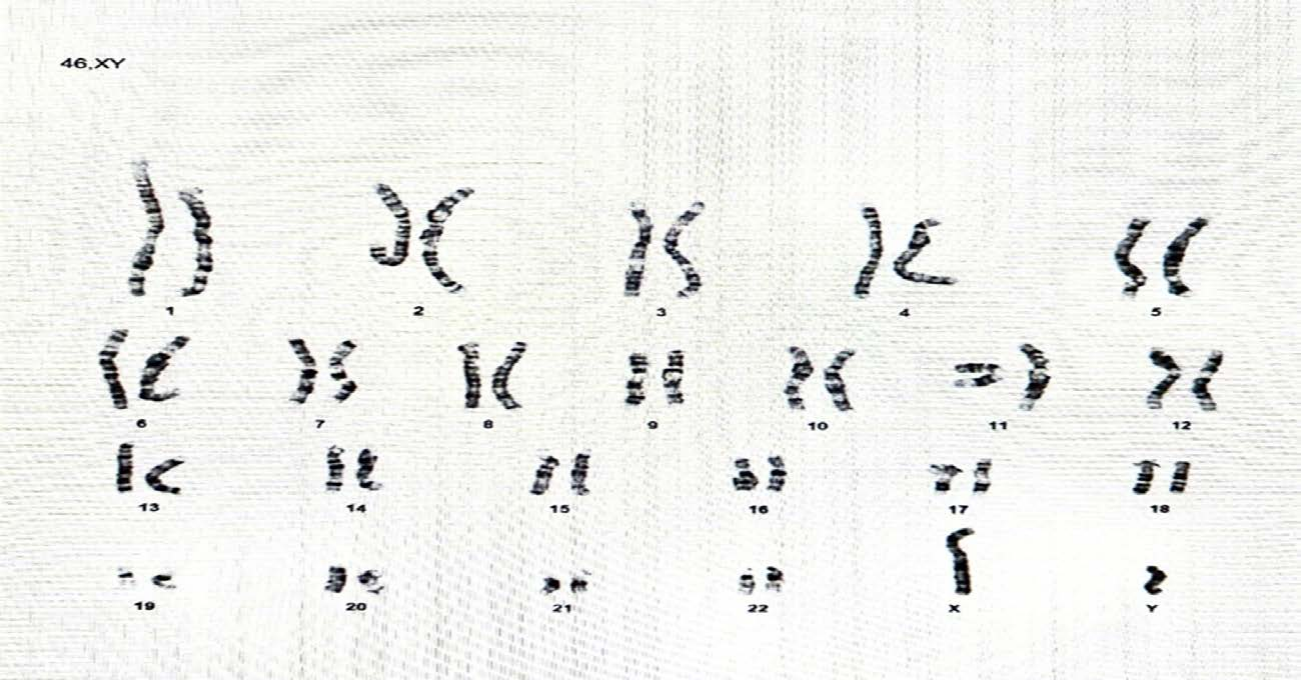
Karyotyping was performed that revealed a male chromosome complement without numerical or structural chromosomal anomalies.

The patient is a male, full-term infant born at 39 weeks gestation, weighing 2.9 kg, with Appearance (skin color), Pulse (heart rate), Grimace (reflex irritability), Activity (muscle tone), and Respiration scores of 8 and 9 at 1 and 5 minutes, respectively. He is the first child of consanguineous Yemeni parents (cousins). The mother, a 25-year-old gravida 1, para 1, has no significant medical history but experienced primary infertility for 5 years but she conceived without medical or assisted reproductive intervention. There is no family history of similar conditions or other genetic disorders. A fetal anomaly ultrasound (US) at 22 weeks gestation revealed short, bowed long bones, suggesting skeletal dysplasia, but no further prenatal investigations were conducted. The infant was stable initially but developed respiratory distress, poor sucking, and an agitated cry at 12 hours of age, requiring neonatal intensive care unit (NICU) admission, nasal oxygen, and orogastric tube (OGT) feeding. At 48 hours, he developed unexplained hyperthermia; a septic workup was negative. Physical examination revealed generalized hypotonia, hyporeflexia, dysmorphic features including fixed joint contractures (Fig. 1A) micrognathia (Fig. 1B), short neck, and camptodactyly with overlapping fingers and toes (Fig. 1D). The infant had short, bowed limbs, and a birth length of 45.5 cm (Fig. 1C). A chest X-ray indicated transient tachypnea of the newborn, and an echocardiogram revealed a patent ductus arteriosus and a small patent foramen ovale, both of which resolved spontaneously. The cranial US was normal, but brain computed tomography showed bilateral periventricular hypodensities (Fig. 2A). A skeletal survey confirmed bilateral symmetrical shortening and bowing of long bones, with cortical thickening, wide metaphases, and decreased bone density (Fig. 2B and C). Karyotyping showed no chromosomal abnormalities (Fig. 3).

Whole exome sequencing, revealed a novel homozygous pathogenic variant in the *LIFR* gene, c.2257dup p.(Arg753Lysfs*20), causing a frameshift mutation starting at codon 753 in exon 16 (Table 1). This variant has not been previously reported in the literature.

**Table 1:** Whole exome sequencing analysis.

Sequence variants							
Gene	Variant coordinates	Amino acid change	SNP identifier	Zygosity	In silico parameters	Allele frequencies	Type and classification*
*LIFR*	NM_001127671.2:c.2257 dup	p.(Arg753Lysfs*20)	N/A	Homozygous	PolyPhen: N/A †Align-GVDG: N/A SIFT: N/A MutationTaster: N/A Conservation_n t: Conservation_a a:	gnomAD: - ESP: - 1000 G: - CentoMD:	Frameshift Pathogenic (class 1)

Variant annotation based on CentoCloud Bioinformatics pipeline.

1000G = 1000Genome project, CentoMD = latest database available, ESP = Exome sequencing project, gnomAD = Genome Aggregation Database, GVDG = Gene Variant Damage/Disruption Grade, N/A = not available/not applicable, PolyPhen = polymorphism phenotyping, SIFT = sorting intolerant from tolerant, SNP = single nucleotide polymorphism.

* Based on ACMG recommendations.

† AlignGVD: C0: least likely to interfere with function, C65: most likely to interfere with function; splicing predictions: Ada and RF scores.

## 3. Hospital course

The patient remained in the NICU for 3 months. Despite several attempts, he could not transition to oral feeding due to poor sucking and swallowing, and the family declined gastrostomy. Hyperthermic episodes occurred 3 to 4 times daily, managed with cold compresses and paracetamol. The infant experienced multiple episodes of respiratory distress requiring varying levels of oxygen therapy. He was discharged home with close follow-up by ophthalmology, orthopedics, genetics, and nutrition clinics. At 5 months, the infant remains on OGT feeding, with a reduced frequency of hyperthermia. His growth parameters at 5 months were a weight of 5 kg (<3rd percentile), length of 53 cm (<3rd percentile), and head circumference of 42.5 cm (25th percentile). The family received genetic counseling regarding the syndrome and its autosomal recessive inheritance pattern, as well as reproductive counseling for future pregnancies, including options for prenatal and preimplantation genetic diagnosis.

## 4. Discussion

SWS was first described by Stüve and Wiedemann^[1]^ in 1971 as a severe, often lethal condition. The syndrome is characterized by a combination of skeletal abnormalities and dysautonomia, which contribute to a high mortality rate in infancy.^[4]^ The underlying cause of SWS is typically a pathogenic variant in the *LIFR* gene, which disrupts the Janus Kinases/Signal Transducer and Activator of Transcription 3 signaling pathway. This disruption impairs both fetal and neonatal bone resorption and neuronal development, leading to the clinical manifestations of SWS.^[2,9]^ A notably high incidence of SWS has been reported in the United Arab Emirates, with an estimated prevalence of 0.5 per 10,000 live births, likely linked to the cultural practice of consanguineous marriage.^[10]^ Similar practices are observed in the Yemeni population, though the exact prevalence of SWS in this group is unknown due to diagnostic challenges, including the syndrome’s genetic and phenotypic variability.^[8]^ In our case, consanguineous marriage was a contributing factor, allowing the homozygous inheritance of an autosomal recessive LIFR variant, which manifested phenotypically as SWS. This pattern of inheritance is consistent with findings from a recent meta-analysis, which reported positive consanguinity in 65% of SWS cases.^[4]^

The initial indication of SWS in our case was detected through the antenatal US, which revealed short, bowed long bones, leading to a provisional diagnosis of skeletal dysplasia. Antenatal US findings have similarly been pivotal in identifying potential SWS cases in other reports.^[4]^ While US is a valuable tool for identifying skeletal dysplasia, it cannot definitively diagnose SWS due to overlapping phenotypic features with other types of dysplasia, such as campomelic and kyphomelic dysplasia.^[11]^ However, advances in antenatal genetic testing now allow for earlier and more accurate diagnosis of SWS, facilitating timely intervention for complications like primary pulmonary hypertension.^[12]^ Catavorello et al^[13]^ documented a case where SWS was suspected based on antenatal US findings and later confirmed through genetic testing.

The morbidity and mortality associated with SWS are largely due to dysautonomia, particularly in the early months of life. In our case, respiratory distress and feeding difficulties necessitated admission to the NICU, along with oxygen therapy and OGT feeding. Respiratory failure, often exacerbated by pulmonary hypertension, remains the leading cause of death in patients with SWS.^[12,14]^ Worsening respiratory distress during infancy can require intensive care, with both invasive and noninvasive respiratory support.^[4]^ Feeding difficulties, particularly poor sucking and swallowing due to pharyngoesophageal dyskinesia, are also well-documented.^[15]^ Our patient required OGT feeding starting on the first day of life, which continued for 5 months. Despite multiple attempts, transitioning to oral feeding was unsuccessful, and the family opted against gastrostomy, instead receiving training on OGT insertion before discharge. Gastrostomy is an important measure to prevent aspiration pneumonia, a significant risk in these patients.^[16]^ There is potential for eventual oral feeding, as other reports have noted successful transitions at 2 to 3 years of age.^[13,15]^

Unexplained hyperthermia, despite negative comprehensive septic workups, was a major concern that prolonged our patient’s NICU stay. This episodic hyperthermia, caused by dysregulation of the body’s temperature control centers, is a common and serious concern in SWS, particularly because of its association with sudden death.^[17]^ Management of hyperthermia is symptomatic, relying on cold compresses and occasional antipyretics. As observed in our patient, the frequency and severity of hyperthermic episodes typically decrease over time as part of the syndrome’s natural progression.^[4]^

Skeletal deformities, including micrognathia, camptodactyly with overlapping fingers and toes, and short, bowed limbs with limited joint mobility, were noted both antenatally and at birth. These dysmorphic features are consistent with those reported in other SWS cases, such as a Saudi infant who exhibited respiratory distress, feeding difficulties, and hyperthermia as additional signs of dysautonomia.^[16]^ The presence of short, bowed limbs is a hallmark of SWS, observed in all documented cases and integral to the syndrome’s definition.^[18]^ Camptodactyly, often accompanied by joint contractures, is another common orthopedic finding.^[4,11]^ Micrognathia, observed in approximately 41% of reported cases, further complicates oral feeding.^[4]^

Our patient demonstrated appropriate neurocognitive development but exhibited significant gross motor delays due to generalized hypotonia. This outcome aligns with the majority of SWS cases, where cognitive function is typically preserved despite other physical challenges.^[4]^ Early intervention with orthopedic and physiotherapy services is crucial to mitigate the impact of hypotonia and skeletal deformities, potentially reducing the need for wheelchair use.^[19]^ In addition, special consideration must be given to the risk of malignant hyperthermia during general anesthesia, as patients with SWS may have an elevated risk for this complication.^[20]^

Although long-term survival in SWS has been reported,^[6]^ the overall prognosis remains poor, especially in the first year of life. The primary causes of mortality include respiratory insufficiency and fatal hyperthermic episodes, with pulmonary hypertension and central adrenal insufficiency also contributing to early fatalities.^[16,21]^

Detailed case descriptions of this rare genetic disorder are essential to enhance our understanding of its genetic and phenotypic variability, ultimately improving diagnosis, management, and patient outcomes. This case report contributes to the growing body of knowledge on SWS and may help address some of the unanswered questions surrounding this syndrome.

Our case parents are Yemeni family who live in Kingdom of Saudi Arabia and practice private jobs with a lack of high-level medical insurance. Economic obstacles prevent further testing for perinatal diagnosis. Cultural and economic reasons were associated with the inability to perform detailed genetic testing of the family to evaluate the carrier status and determine the recurrence risk.

## 5. Conclusion

SWS is a severe autosomal recessive disorder characterized by skeletal dysplasia and dysautonomia. Early signs include feeding difficulties, respiratory distress, and hyperthermia, with skeletal dysplasia aiding in diagnosis. Genetic testing is essential for confirmation. Although SWS is associated with high infant mortality, improved care has led to increased survival in some cases. Management remains largely supportive, focusing on preventing aspiration, managing hyperthermia, and addressing orthopedic issues. Prenatal genetic testing should be offered to families with a history of consanguinity for early diagnosis and better management of future pregnancies.

## Acknowledgments

The authors express their thanks to the patient and his family for their contribution to the current study. The authors would like to thank the Research, Development, and Innovation Authority (RDIA) for supporting this work.

## Author contributions

**Data curation:** Hanan Sakr Sherbiny, Jaber A. Alfaifi, Anees Obaid Ahmed, Hany Hassan, Raydaa Abdullah.

**Formal analysis:** Hanan Sakr Sherbiny, Mohammed A. M. Oshi, Naglaa M. Kamal.

**Funding acquisition:** Hanan Sakr Sherbiny.

**Investigation:** Hanan Sakr Sherbiny, Jaber A. Alfaifi.

**Methodology:** Hanan Sakr Sherbiny.

**Project administration:** Hanan Sakr Sherbiny.

**Supervision:** Hanan Sakr Sherbiny, Jaber A. Alfaifi.

**Validation:** Hanan Sakr Sherbiny.

**Visualization:** Hanan Sakr Sherbiny.

**Writing – original draft:** Hanan Sakr Sherbiny, Jaber A. Alfaifi, Anees Obaid Ahmed, Hany Hassan, Raydaa Abdullah, Mohammed A. M. Oshi, Shaher A. Alsuwat, Raghad M. Al-Juaid, Aseel A. Neyaz, Naglaa M. Kamal.

**Writing – review & editing:** Hanan Sakr Sherbiny, Jaber A. Alfaifi, Mohammed A. M. Oshi, Shaher A. Alsuwat, Raghad M. Al-Juaid, Aseel A. Neyaz, Naglaa M. Kamal.
